# The Durable Effect of Pyrotinib Plus Trastuzumab and Chemotherapy in HER2-Positive Gastric Cancer With Brain Metastases: A Case Report and Literature Review

**DOI:** 10.3389/fonc.2022.940263

**Published:** 2022-07-08

**Authors:** Xinwei Wang, Yun Zeng, Junling Zhang, Mengli Huang, Bijian Yin

**Affiliations:** ^1^ Department of Medical Oncology, Jiangsu Cancer Hospital, Jiangsu Institute of Cancer Research, The Affiliated Cancer Hospital of Nanjing Medical University, Nanjing, China; ^2^ Medical Department, 3D Medicines Inc., Shanghai, China

**Keywords:** HER-2, gastric cancer, pyrotinib, trastuzumab, case report

## Abstract

Gastric cancer (GC) is a disease with macromolecular phenotypic heterogeneity and poor prognosis, especially for metastatic GC (mGC). Chemotherapy is the first choice for second-line treatment. However, the benefits of second-line chemotherapy are limited, so there is an urgent need for new treatment regimens to improve patient outcomes. A 65-year-old man with mGC was HER-2 positive. Standard trastuzumab, combined with chemotherapy, was given in the first-line therapy and progression-free survival (PFS) was 8 months. Second-line treatment with pyrotinib in combination with trastuzumab and chemotherapy yielded a PFS of 20 months, in sharp contrast to a median survival of 2.9-6.2 months for a majority of advanced GC patients. This case provides a meaningful reference for the second-line treatment of mGC patients with HER-2 positive. This case also provides valuable information on the response to pyrotinib plus trastuzumab of patients with brain metastases and a better understanding of dual target combination therapy applications in the future.

## Introduction

Gastric cancer (GC) is the fifth most common cancer in the world, with nearly 1.09 million new cases in 2020. In addition, GC is the fourth leading cause of cancer death, with nearly 770,000 deaths worldwide in 2020 (1). China has a high incidence of GC, accounting for 43.9% of the global incidence and 48.6% of the global mortality (2). Different from other countries, about 79.6% of Chinese patients with GC were locally advanced and metastatic GC (mGC), and the 5-year overall survival (OS) rate was only 9.4% in patients with mGC (3, 4).

The human epidermal growth factor receptor 2 (HER2) is overexpressed or amplified in 6%-36% of GC and is an important target for mGC treatment strategies (5). The ToGA study was the first to demonstrate that trastuzumab combined with chemotherapy significantly prolonged median OS (mOS) in patients with advanced HER2-positive GC (16.0 vs. 11.8 months, HR, 0.65, 95%Cl 0.51-0.83, *P*=0.036) (6). Similar results have been found based on real-world data from China (7). However, there are few treatment options for mGC after first-line treatment failure. In randomized trials, selected second-line chemotherapy significantly improved OS compared to the best supportive treatment, but the mOS was less than 6 months (8–10). Although ramucirumab plus paclitaxel provided a significantly longer OS than the placebo plus paclitaxel (mOS: 8.71 months vs 7.92 months (HR, 0.963, 95%Cl 0.771-1.203, *P*=0.7426) (11), ramucirumab is unfunded for GC in China.

Pyrotinib is a second-generation, irreversible, well-absorbed dual human epidermal growth factor receptor 1 and 2 inhibitor (12). The phase 1 study preliminarily demonstrated the safety and antitumor activity of pyrotinib as monotherapy and in combination with capecitabine (13). In HER2-positive locally relapsed or metastatic breast cancer following prior trastuzumab and taxanes, pyrotinib plus capecitabine provided significantly longer progression-free survival (PFS) than placebo plus capecitabine (median PFS: 11.1 months vs 4.1 months, HR, 0.18, 95%Cl 0.13-0.26, *P*<0.001). Pyrotinib also extended PFS, even in patients with brain metastases at baseline (median PFS: 6.9 months vs 4.2 months, HR, 0.32, 95%Cl 0.13-0.77, *P*=0.011) (14). Herein, we report a case of HER2-positive mGC with a long-survival benefit from second-line pyrotinib plus trastuzumab and chemotherapy after first-line treatment failed.

## Case Presentation

**Figure 1:**
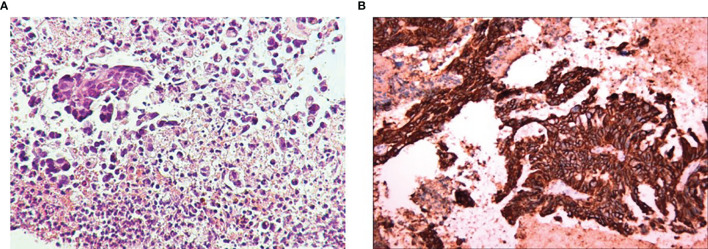
Hematoxylin-Eosin staining **(A)** and immunohistochemical staining of HER2 expression **(B)**.

**Figure 2:**
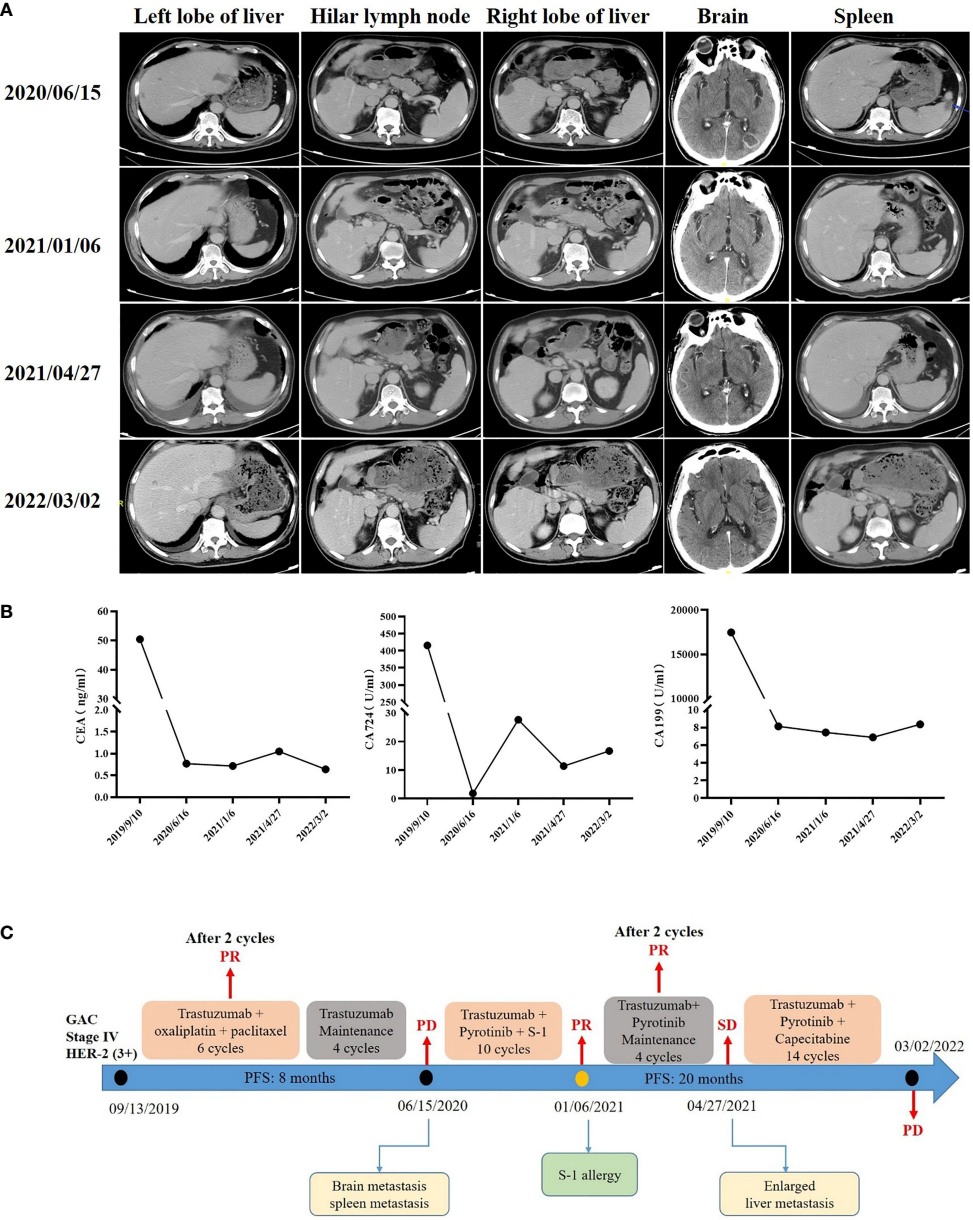
Computed tomography during treatment **(A)**, blood tumor antigen changes **(B)**, and timeline of the clinical course in this patient **(C)**.

A 65-year-old man presented with epigastric discomfort without obvious abdominal pain, diarrhea, or black stools. The patient with EGOC PS 0 had no other past medical history and family history. Endoscopic examination in another hospital showed that the anterior pyloric area of gastric antrum was occupied with low adhesion carcinoma. Abdominal computerized tomography (CT) showed thickening of the gastric wall in the antrum and low density of the liver and hilar lymph nodes (left lobe of liver, 4.21×4.18 cm; right lobe of liver, 4.16×3.36 cm; hilar lymph node, 1.48×1.43 cm). Routine biopsy showed a poorly differentiated adenocarcinoma (TxNxM1, stage IV, **Figure 1A**). Immunohistochemistry studies revealed HER2 positive (3+, **Figure 1B**). In addition, laboratory examinations revealed elevations in the levels of carbohydrate antigen 19-9 (CA19-9; 17463 U/ml), carbohydrate antigen 724 (CA724; 415.5 U/ml) and carcinoembryonic antigen (CEA; 50.41 ng/ml). He underwent 6 cycles of combination therapy composed of trastuzumab plus oxaliplatin and paclitaxel. Liver metastatic lesions (left lobe of liver, 2.66×2.12 cm; right lobe of liver, 2.59×1.56 cm) and hilar lymph node (1.24×0.97 cm) were shrunk, so that the efficacy was assessed as partial response (PR) according to iRECIST criteria (**Figure 2A**), followed by maintenance therapy with trastuzumab. After 4 cycles, although no abnormalities were observed in CEA, CA19-9, or CA724, CT showed spleen metastasis **(**1.34×1.23 cm**)** and brain metastasis (2.06×1.60 cm), with the efficacy defined as progressive disease (PD) (**Figure 2A**).

In second-line therapy, the patient received trastuzumab plus pyrotinib and S-1 for 10 cycles, with the efficacy defined as PR. However, CA724 (27.6 U/ml) was significantly elevated (**Figure 2B**). S-1 was suspended due to an allergy to S-1. However, considering the clinical benefits, only trastuzumab plus pyrotinib continued to be used. After 4 cycles, an abdominal CT scan identified a slight enlargement of liver metastases (left lobe of liver mass from 2.4×1.18 cm to 2.62×1.52 cm). The efficacy was evaluated as stable disease (SD) (**Figure 2A**). The patient started to receive trastuzumab plus pyrotinib and capecitabine since April 27, 2021. At a recent re-examination, CT showed an increased left lobe of liver mass (3.23×1.71 cm) with effect assessed as PD (**Figure 2A**). Eventually second-line treatment of PFS of up to 20 months was given (**Figure 2C**).

## Discussion

As far as we know, this is the first report of HER2 positive mGC response to pyrotinib plus trastuzumab and chemotherapy with a long survival time. Our case emphasizes the importance of dual target combination therapy for patients with advanced second-line GC.

GC is a disease with high molecular and phenotypic heterogeneity, among which HER2 positive GC is one of the special molecular subtypes. Based on the TOGA study, patients with HER2 overexpressing GC should be treated with trastuzumab in addition to first-line chemotherapy, followed by trastuzumab monotherapy as maintenance. In our case, the patient achieved PR after 6 cycles of first-line trastuzumab plus chemotherapy, followed by trastuzumab maintenance and first-line PFS for 8 months.

In second-line therapy, chemotherapy monotherapy (paclitaxel/docetaxel/irinotecan) is the preferred treatment for HER2-positive GC patients in China. However, the benefits of second-line chemotherapy are limited. In clinical trials, the mOS for patients receiving second-line chemotherapy was about 6 months (9, 10). Previous clinical trials of second-line targeted treatment for mGC failed (**Table 1**). In this case, after communication with the patient and his family, pyrotinib plus trastuzumab and chemotherapy were given, the efficacy was evaluated as PR and the final second-line PFS was more than 20 months, significantly higher than 2.9-6.2 months with chemotherapy (19). The treatment of HER2-positive GC still needs to be optimized continuously, including exploration of the combination of different treatment options, cross-line therapy, and maintenance therapy.

**Table 1:** The previous clinical trials of second-line treatment for mGC.

Trial	Phase	Tumor type	Treatment settings	Primary outcome
T-ACT (15)	II	HER2-Positive advanced gastric or gastroesophageal junction cancer	Paclitaxel vs paclitaxel with trastuzumab	mPFS: 3.2 vs 3.7 months (*P* = .33)
Horita Y et al. (16)	II	HER2 positive gastric adenocarcinoma	Paclitaxel plus trastuzumab	ORR: 21.4% (expected ORR=35%)
TyTAN (17)	III	HER2-amplified advanced GC	Lapatinib plus paclitaxel vs paclitaxel	mOS: 11.0 vs 8.9 months (*P* = .1044)
GATSBY (18)	II/III	HER2-positive locally advanced or metastatic gastric/gastroesophageal junction adenocarcinoma	Trastuzumab emtansine vs taxane	mOS: 7.9 vs 8.6 months (*P* = 0.86)

In summary, this case is reported in only one person, but shows that pyrotinib plus trastuzumab and chemotherapy are effective and tolerated in HER2-positive mGC with brain metastases. Therefore, for patients with HER2-positive mGC with brain metastases, the treatment strategy of dual-target combination therapy deserves further study in large-scale clinical trials to improve the prognosis of these patients.

## Data Availability Statement

The original contributions presented in the study are included in the article/supplementary material. Further inquiries can be directed to the corresponding author.

## Ethics Statement

The studies involving human participants were reviewed and approved by Jiangsu Cancer Hospital. The patients/participants provided their written informed consent to participate in this study. Written informed consent was obtained from the individual(s) for the publication of any potentially identifiable images or data included in this article.

## Author Contributions

XW, JZ and BY designed the study. XW and JZ wrote the first draft of the manuscript. XW and YZ treated the patients and acquired data. All authors analyzed the data. XW, JZ and BY interpret the data. All authors contributed to the article and approved the submitted version.

## Conflict of Interest

JZ and MH are employees of 3D Medicines Inc.

The remaining authors declare that the research was conducted in the absence of any commercial or financial relationships that could be construed as a potential conflict of interest.

## Publisher’s Note

All claims expressed in this article are solely those of the authors and do not necessarily represent those of their affiliated organizations, or those of the publisher, the editors and the reviewers. Any product that may be evaluated in this article, or claim that may be made by its manufacturer, is not guaranteed or endorsed by the publisher.

## Abbreviations

GC, gastric cancer; mGC, metastatic GC; PFS, progression-free survival; HER2, Human epidermal growth factor receptor 2; OS, overall survival; mOS, median OS; CT, computerized tomography; CEA, carcinoembryonic antigen; CA199, carbohydrate antigen 19-9; CA724, carbohydrate antigen 724; PR, partial response; PD, progressive disease; SD, stable disease.
